# CD45Rb-low effector T cells require IL-4 to induce IL-10 in FoxP3 Tregs and to protect mice from inflammation

**DOI:** 10.1371/journal.pone.0216893

**Published:** 2019-05-23

**Authors:** Mark B. Jones, Carlos A. Alvarez, Jenny L. Johnson, Julie Y. Zhou, Nathan Morris, Brian A. Cobb

**Affiliations:** 1 Department of Pathology, Case Western Reserve University School of Medicine, Cleveland, Ohio, United States of America; 2 Department of Population and Quantitative Health Sciences, Case Western Reserve University School of Medicine, Cleveland, Ohio, United States of America; Université Paris Descartes, FRANCE

## Abstract

CD4^+^ effector/memory T cells (Tem) represent a leading edge of the adaptive immune system responsible for protecting the body from infection, cancer, and other damaging processes. However, a subset of Tem cells with low expression of CD45Rb (Rb^Lo^Tem) has been shown to suppress inflammation despite their effector surface phenotype and the lack of FoxP3 expression, the canonical transcription factor found in most regulatory T cells. In this report, we show that Rb^Lo^Tem cells can suppress inflammation by influencing Treg behavior. Co-culturing activated Rb^Lo^Tem and Tregs induced high expression of IL-10 *in vitro*, and conditioned media from Rb^Lo^Tem cells induced IL-10 expression in FoxP3^+^ Tregs *in vitro* and *in vivo*, indicating that Rb^Lo^Tem cells communicate with Tregs in a cell-contact independent fashion. Transcriptomic and multi-analyte Luminex data identified both IL-2 and IL-4 as potential mediators of Rb^Lo^Tem-Treg communication, and antibody-mediated neutralization of either IL-4 or CD124 (IL-4Rα) prevented IL-10 induction in Tregs. Moreover, isolated Tregs cultured with recombinant IL-2 and IL-4 strongly induced IL-10 production. Using house dust mite (HDM)-induced airway inflammation as a model, we confirmed that the *in vivo* suppressive activity of Rb^Lo^Tem cells was lost in IL-4-ablated Rb^Lo^Tem cells. These data support a model in which Rb^Lo^Tem cells communicate with Tregs using a combination of IL-2 and IL-4 to induce robust expression of IL-10 and suppression of inflammation.

## Introduction

Regulatory T cells (Tregs) are critical for the maintenance of immune homeostasis. The most widely recognized and studied subset of Tregs express the transcription factor FoxP3 and can be induced peripherally or develop directly in the thymus [1–3]; however, FoxP3^-^ type 1 regulatory cells (Tr1) are also well-characterized [4, 5]. Another CD4^+^ T cell subset known to have regulatory/suppressive properties are those lacking FoxP3 while expressing low concentrations of the activation marker CD45Rb (Rb^Lo^) at the cell surface. These Rb^Lo^ T cells inhibit the induction of wasting disease in SCID mice [6], type 1 diabetes [7], a plant antigen-based model of asthma [8], and the formation of adhesions [9]. In agreement with these reports, we recently found that the polysaccharide antigen PSA from *Bacteroides fragilis* significantly decreased susceptibility to the development of pulmonary inflammation through activation and expansion of CD4^+^FoxP3^-^CD45Rb^Lo^ effector-memory (CD62L^-^CD44^+^) T cells (Rb^Lo^Tem)[10–12].

Rb^Lo^Tem cells are known to depend upon IL-10 for their protective efficacy [13, 14]. Consistent with this, we found that the suppressive activity of Rb^Lo^Tem cells required IL-10 in both humans *in vitro* [15] and mice *in vivo* [10, 12]. In an *in vivo* model in which all cells lacked IL-10, the Rb^Lo^Tem cells failed to protect the animals from pulmonary inflammation [10]. However, reciprocal adoptive transfer experiments in which activated wild type (WT) or IL-10-deficient (IL-10^-/-^) Rb^Lo^Tem cells were given to WT or IL-10^-/-^ recipients, we discovered that IL-10 was dispensable in the Rb^Lo^Tem cells but not in the recipient [12]. Moreover, adoptive transfer of IL-10^-/-^ Rb^Lo^Tem cells induced IL-10 expression in CD4^+^FoxP3^+^ Tregs in the lung [12], suggesting a model in which Rb^Lo^Tem cells suppress inflammation by the selective induction of IL-10 in FoxP3^+^ Tregs *in vivo* through an unknown mechanism.

In this study, we report the discovery of a mechanism by which Rb^Lo^Tem cells communicate with and drive suppressive activity of FoxP3^+^ Tregs to regulate inflammation. Consistent with our *in vivo* studies [12], co-cultured Rb^Lo^Tem cells induced FoxP3^+^ Tregs to secrete high concentrations of IL-10 *in vitro*. Conditioned media from activated Rb^Lo^Tem cells also induced IL-10 in FoxP3^+^ Tregs both *in vivo* and *in vitro*, demonstrating cell-contact independence in the communication pathway between these cells. Deep sequencing transcriptomics of activated Rb^Lo^Tem cells versus CD45Rb^Hi^ T naïve cells identified potential soluble mediators, and antibody-mediated neutralization experiments suggested that both IL-2 and IL-4 were necessary for IL-10 induction in Tregs. Supplementation of Treg media with recombinant IL-2 and IL-4 confirmed this *in vitro*, while the use of IL-4-deficient Rb^Lo^Tem cells showed a lack of protective efficacy in a model of pulmonary inflammation when compared to WT cells. These results reveal an intrinsic IL-2 and IL-4-dependent T cell crosstalk network connecting the suppressive capacity of Rb^Lo^Tem cells with canonical FoxP3^+^ Tregs, which could potentially be harnessed for the treatment of inflammation-mediated diseases.

## Materials and methods

### Mice

C57BL/6 (Stock #000664), IL-10-eGFP (B6.129S6-Il10^*tm1Flv*^/J, Stock #008379), IL-10-null (B6.129P2-*Il10*^*tm1Cgn*^/J, Stock #002251), FoxP3-RFP (C57BL/6-FoxP3^tm1Flv^/J), and IL-4-knockout (B6.129P2-*Il4*^*tm1Cgn*^/J) mice, all on the C57BL/6 background, were purchased from the Jackson Laboratory (Bar Harbor, ME). FoxP3-eGFP animals were a kind gift of Drs. Rudensky and Letterio. Mice were fed standard chow (Purina 5010) on a 12-hour light/dark cycle in a specific pathogen free facility. Enrichment and privacy provided in mating cages by ‘love shacks’ (Bio Serv 53352–400). Mouse studies, and all animal housing at Case Western Reserve University were approved by and performed according to the guidelines established by the Institutional Animal Care and Use Committee of CWRU.

### Primary splenic T cells

Primary splenocytes were isolated from freshly harvested spleens, and reduced to a single cell suspension by passing them through a sterile 100μM nylon mesh cell strainer (Fisher Scientific, Hampton, NH). For splenic T cell enrichment, single cell suspensions were labeled with anti-mouse CD4 magnetic microbeads, or alternatively negatively selected to yield untouched CD4 cells, and positively selected for CD25 by a mouse regulatory T cell isolation kit (Miltenyi Biotec, San Diego, CA), and separated with an AutoMACS Pro Separator (Miltenyi Biotec, San Diego, CA), per manufacturer’s instructions.

### Pulmonary inflammation and adoptive transfer

#### HDM model

Mice were challenged with house dust mite antigen (HDM, *D*. *Farinae*, GREER, Lenoir, NC) by intranasal delivery of 20μg HDM/dose in PBS on days 0–4 and 7–11 and sacrificed on day 14 [16]. For adoptive transfer, 60,000 Rb^Lo^Tem cells were harvested by FACS from FoxP3-eGFP reporter mice and i.v. injected on day 6. Animals were anesthetized with 3% isoflurane (Baxter) with an anesthesia system (VetEquip, Livermore, CA) for intranasal administration. Euthanasia, BALf recovery, and lung tissue preparation was performed as previously reported [10, 12]. BALf automated differentials were acquired by a HemaVet 950 Hematology Analyzer.

### Flow cytometry and cell sorting

For splenic T cell sorting, magnetic bead-mediated positively selected CD4^+^ cells were stained with combinations of antibodies (0.5μg/mL per) to CD62L-PE (BioLegend, San Diego, CA) or CD62L-BB515 (BD Bioscience), CD44-APC (BioLegend, San Diego, CA), CD45Rb-APC/Cy7 (BioLegend, San Diego, CA), CD124 (1μg/mL, BD Biosciences, San Jose, CA), and CD25-BV421 (BD Biosciences, San Jose, CA). For Tregs, FoxP3-RFP reporter signal was used for FACS. Cells were washed twice in MACS buffer (Miltenyi Biotec, San Diego, CA) before sterile cell sorting using a FACSAria (BD Biosciences, San Jose, CA) with the support of the Cytometry & Imaging Microscopy Core Facility of the Case Comprehensive Cancer Center. Analysis of all FACS data was performed using FlowJo v10 (Tree Star, Inc., Ashland, OR).

### Cell culture

**Fig 1:**
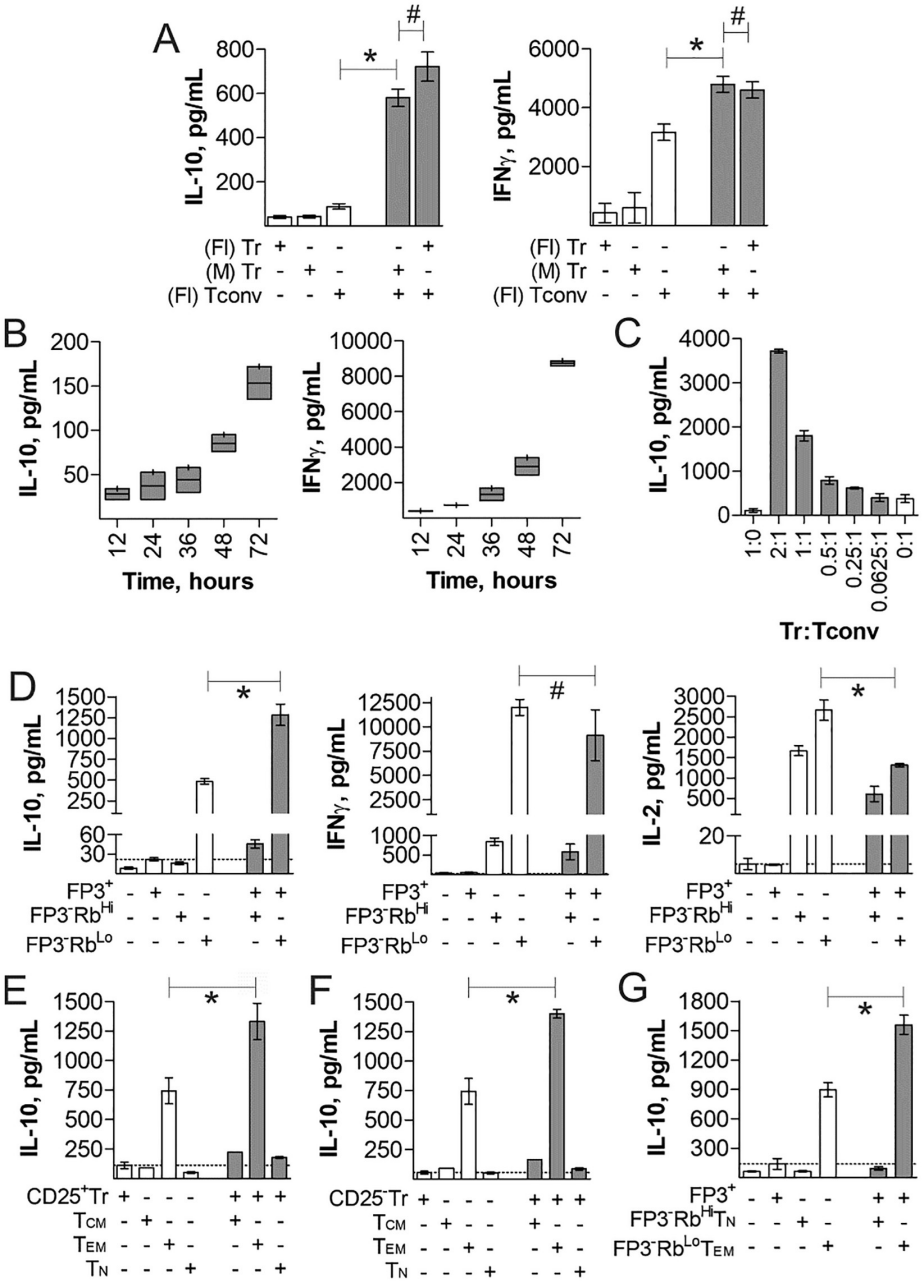
IL-10 production by Tregs is synergistically enhanced by CD4^+^FoxP3^-^CD45Rb^Lo^CD44^+^CD62L^-^ T cells. Tregs and various CD4^+^ T cell subsets were cultured *in vitro* with plate-bound anti-CD3ε antibody for 3 days, unless otherwise specified, to measure their cytokine responses by ELISA. (A) Comparison of mono- and co-cultures of magnetic bead purified (M) CD4^+^ Tconv and CD4^+^CD25^+^ Tr cells vs. flow sorted (Fl) CD4^+^CD25^+^ Tr cells. (B) Time course of cytokine production from co-cultures of flow-sorted Tconv and CD25^+^ Tregs. (C) Co-cultures of flow-sorted 50k Tconv and varied Tregs at indicated ratios. (D) 1:1 Cultures of flow sorted CD4^+^FoxP3^+^ Tregs and CD4^+^FoxP3^-^CD45Rb^Hi/Lo^ cells, showing IL-10, IFNγ, and IL-2 production by ELISA. (E) 1:1 cultures of CD4^+^CD25^+^FoxP3^+^ Tregs and CD4^+^CD25^-^FoxP3^-^CD62L^+^CD44^+^ (Tcm), CD4^+^CD25^-^FoxP3^-^CD62L^-^CD44^+^ (Tem), and CD4^+^CD25^-^FoxP3^-^CD62L^+^CD44^-^ (Tn) cells. (F) 1:1 cultures of CD4^+^CD25^-^FoxP3^+^ Tregs and Tcm, Tem, or Tn cells. (G) 1:1 cultures of CD4^+^FoxP3^+^ Tregs and CD4^+^FoxP3^-^CD45Rb^Lo^CD62L^-^CD44^+^ (Rb^Lo^Tem) or CD4^+^FoxP3^-^ CD45Rb^Hi^CD62L^+^CD44^-^ (Rb^Hi^Tn) cells. * = p<0.05; # = p>0.05. P value calculated from Student’s T-Test. Error bars show mean with SEM. For A-B and D-F, n = 3 experiments. For C, n = 6 experiments. For G, n = 4 experiments.

After flow sorting, cells were cultured in 96-well plates (Corning, Corning, NY) at 50,000 cells per type per well in advanced RPMI (Gibco/Fisher Scientific, Waltham, MA) supplemented with 5% Australian-produced heat-inactivated fetal bovine serum, 55μM β-mercaptoethanol, 100U/mL and 100μg/mL Penicillin/Streptomycin, and 0.2mM L-glutamine (Gibco/Fisher Scientific, Waltham, MA) at 5% CO_2_, 37°C. For activating conditions, wells were coated with αCD3ε (eBioscience, San Diego, CA) at 2.5μg/mL in PBS then incubated at 37°C for 4 hours followed by two washes with PBS before receiving cells. For fixation studies, indicated cell types were resuspended in 2% paraformaldehyde in PBS for 10 minutes on ice. Cells were washed twice in PBS before being co-cultured with live CD4^+^FoxP3^+^ cells. All co-culture experiments were performed at 1:1 cell ratios, except for the experiments in Fig 1C, which were performed by maintaining constant Tconv cell numbers (50k) and altering the number of Tregs relative to the 50k Tconv, as indicated.

### ELISA, blocking, supplementation and Luminex

Cytokine levels were analyzed by standard sandwich ELISA performed as per manufacturer’s instructions (BioLegend, San Diego, CA), modified to utilize europium-conjugated streptavidin (Perkin-Elmer), and detected with a Victor V3 plate reader (Perkin Elmer, San Jose, CA). Blocking experiments utilized antibodies to IFNγ (10μg/mL, BioLegend, San Diego, CA), IL-21 (10μg/mL, eBioscience, San Diego, CA), IL-22 (10μg/mL, eBioscience), CSF2 (10μg/mL, eBioscience), IL-9 (1μg/mL, eBioscience), IL-13 (2μg/mL, eBioscience), IL-4 (1μg/mL, eBioscience), IL-24 (2μg/mL, eBioscience), IL-3 (1μg/mL, eBioscience), Neuropilin-1 (1μg/mL, R&D Systems, Minneapolis, MN), CD124 (1μg/mL, BD Biosciences, San Jose, CA), and corresponding isotype controls IgG1 and IgG2a (BioLegend, San Diego, CA). For CD124 blocking experiments, indicated cell types were incubated with 1μg/mL αCD124 at 4°C for 15 minutes, washed twice with PBS, then combined into co-culture. Supplementation assays were performed with recombinant mouse IL-2 and IL-4 (R&D Systems, Minneapolis, MN) at the indicated concentrations. For Luminex assays, media from indicated cultured populations were snap frozen in liquid nitrogen and sent to Eve Technologies (Calgary, Ontario, Canada) for mouse 32-plex and TGFβ 3-plex analysis.

### RNAseq and analysis

For RNA quantitation, CD4^+^FoxP3^+^ (Treg), CD4^+^FoxP3^-^CD45Rb^Hi^CD62^Hi^CD44^Lo^ (Rb^Hi^Tn), CD4^+^FoxP3^-^CD45Rb^Lo^CD62L^Lo^CD44^Hi^ (Rb^Lo^Tem) cells were flow sorted as above, pelleted and snap frozen in liquid nitrogen. RNA extraction and RNAseq was performed at Ambry Genetics. Initial processing of the data was performed by using the trimmomatic [17] program to trim the poor quality reads, and fastQC to investigate read quality. TopHat [18] was used to align the reads to the “mm10” genome guided by the Gencode vm4 [19] annotations. To count the number of reads aligning to each feature in Gencode vm4, we used htseq-count [20]. RPKM values were calculated in R, and the differential expression analysis between “Hi” and “Lo” samples was performed using the negative binomial test implemented in DEseq2 [21].

### Histology and microscopy

Tissues were blocked, sectioned, and stained with H&E by the Case Western Reserve University Tissue Procurement and Histology Core Facility, and at AML Laboratories, Inc. (Jacksonville, FL). Unstained sections were stained with rat-anti-EpCAM-AF488 (eBioscience, San Diego, CA) or rat-anti-EpCAM-AF594 (BioLegend, San Diego, CA) at 6μg/mL, and rabbit-anti-myeloperoxidase (1:100, Abcam, Cambridge, MA) then either anti-rabbit-APC (Thermo/Fisher, Waltham, MA) or anti-rabbit-AF488 (Jackson ImmunoResearch, West Grove, PA) at 1:1000. Confocal analysis and imaging was performed on a Leica SP5 confocal microscope; H&E images were acquired with a Leica DM IL LED.

### Clinical scoring

Scoring of H&E-stained lung tissues was performed by Dr. Cobb in a blinded fashion based on the rubric shown in Table 1, with a maximum score of 9 characterized by severe airway epithelial hyperplasia and large numbers of infiltrating cells disseminated throughout the tissue.

**Table 1:** Lung inflammation clinical score rubric.

Parameter	Score	Description
Hyperplasia	0	None
	1	Not more than 2 bronchi with mild to moderate epithelial hyperplasia
	2	Many bronchi with moderate hyperplasia
	3	Most bronchi with moderate to severe hyperplasia
Infiltration	0	None
	1	Small numbers of infiltrating immune cells (undifferentiated)
	2	Moderate numbers of infiltrating immune cells
	3	High numbers of infiltrating immune cells
Localization	0	Infiltrating cells limited to one or two bronchi
	1	Infiltrating cells seen around a plurality of bronchi
	2	Infiltrating cells seen around all bronchi and some dissemination in the alveolar space
	3	Infiltrating cells throughout the tissue

H&E stained lung sections were scored for epithelial cell hyperplasia, infiltration of immune cells, and localization of immune cells on a zero to 3 scale for each parameter. Final clinical scores were the sum of each parameter for each tissue.

### Data analyses

All data are represented by mean ± SEM from at least 3 independent experiments, with the exception of the RNAseq, which was performed in duplicate for each subset. Pulmonary inflammation data sets include a minimum of four, but more commonly six animals per group per experiment. Data and statistical measurements were generated with GraphPad Prism (v5.0). For comparisons between two groups, Student’s *t*-test was used; comparisons between multiple groups utilized analysis of variance.

## Results

### Rb^Lo^Tem cells induce IL-10 in FoxP3^+^ Tregs

Our previous studies revealed that conventional CD4^+^ T cells activated by the polysaccharide PSA from the capsule of *Bacteroides fragilis* can influence FoxP3^+^ Tregs to produce IL-10 through an unknown pathway [10, 12]. Here, we investigated the ability of CD4^+^ T cells to influence Tregs and their production of IL-10 in the absence of PSA. CD4^+^CD25^+^ Tregs and CD4^+^CD25^-^ Tconv cells were isolated from WT mice using either magnetic beads (M) or sterile flow sorting (Fl), and stimulated with anti-CD3ε antibody alone or in co-culture for different amounts of time. Despite having not been previously exposed to PSA, we found that when cultured together, Tregs and Tconv cells synergistically produced IL-10 at 72 hours, while IFNγ production was simply additive (Fig 1A and 1B). Moreover, variations of the ratio of Treg:Tconv demonstrates that the amount of IL-10 is proportional to the number of Tregs, not the number of Tconv cells (Fig 1C).

PSA exposure expands a CD4^+^FoxP3^-^CD45Rb^Lo^ T cell population in both humans [15] and mice [10, 11], and this population was found to induce IL-10 production by lung-localized FoxP3^+^ Tregs [12]. We therefore isolated CD4^+^FoxP3^-^CD45Rb^Lo^ (FoxP3^-^Rb^Lo^), CD4^+^FoxP3^-^CD45Rb^Hi^ (FoxP3^-^Rb^Hi^), and CD4^+^FoxP3^+^ Tregs (FoxP3^+^) from PSA-naïve FoxP3-eGFP reporter mice and stimulated them as before with anti-CD3ε. We found that while the FoxP3^-^Rb^Lo^ subset produced some IL-10 alone, the IL-10 concentration was increased over 2 fold in co-culture with FoxP3^+^ cells on day 3 (Fig 1D). IFNγ trended down but was not significant while IL-2 was significantly reduced in co-culture (Fig 1D). Importantly, the FoxP3^-^Rb^Hi^ population, despite their ability to produce IL-2, did not induce significant IL-10 secretion *in vitro*.

To further clarify the identification of T cells responsible for communicating with FoxP3^+^ Tregs, CD4^+^FoxP3^+^ Tregs with or without CD25, CD4^+^ naïve (Tn; CD62L^+^CD44^-^), effector memory (Tem; CD62L^-^CD44^+^) and central memory (Tcm; CD62L^+^CD44^+^) T cells from FoxP3-eGFP mice were isolated and stimulated as before in various combinations. Tem cells were the only subset to produce IL-10 alone, and the only ones to synergistically induce IL-10 in co-culture with Tregs (Fig 1E and 1F), suggesting that the T cell subset responsible for the Treg IL-10 effect is antigen experienced and are most likely CD4^+^FoxP3^-^CD62L^-^CD44^+^CD45Rb^Lo^ (Rb^Lo^Tem) cells. The presence (Fig 1E) or absence (Fig 1F) of the IL-2Rα (CD25) on the Treg population (see S1 Fig for the gating strategy) did not have an impact on the synergistic interaction between these cells. Finally, we isolated Rb^Lo^Tem and CD4^+^FoxP3^-^CD62L^+^CD44^-^CD45Rb^Hi^ (Rb^Hi^Tn) T cells (see S2 Fig for the gating strategy) and compared their ability to induce IL-10 synergy with FoxP3^+^ Tregs. Only the Rb^Lo^Tem cells produced IL-10 alone and synergistically with FoxP3^+^ Tregs (Fig 1G). These findings align the identity of the cells capable of stimulating IL-10 production in Tregs with those which are selectively expanded by PSA [10–12]. More importantly, the data demonstrate that Rb^Lo^Tem cell communication with Tregs is independent of PSA, and is therefore an intrinsic property of this regulatory T cell subset.

### IL-10 synergy is unique among common cytokines

**Fig 2:**
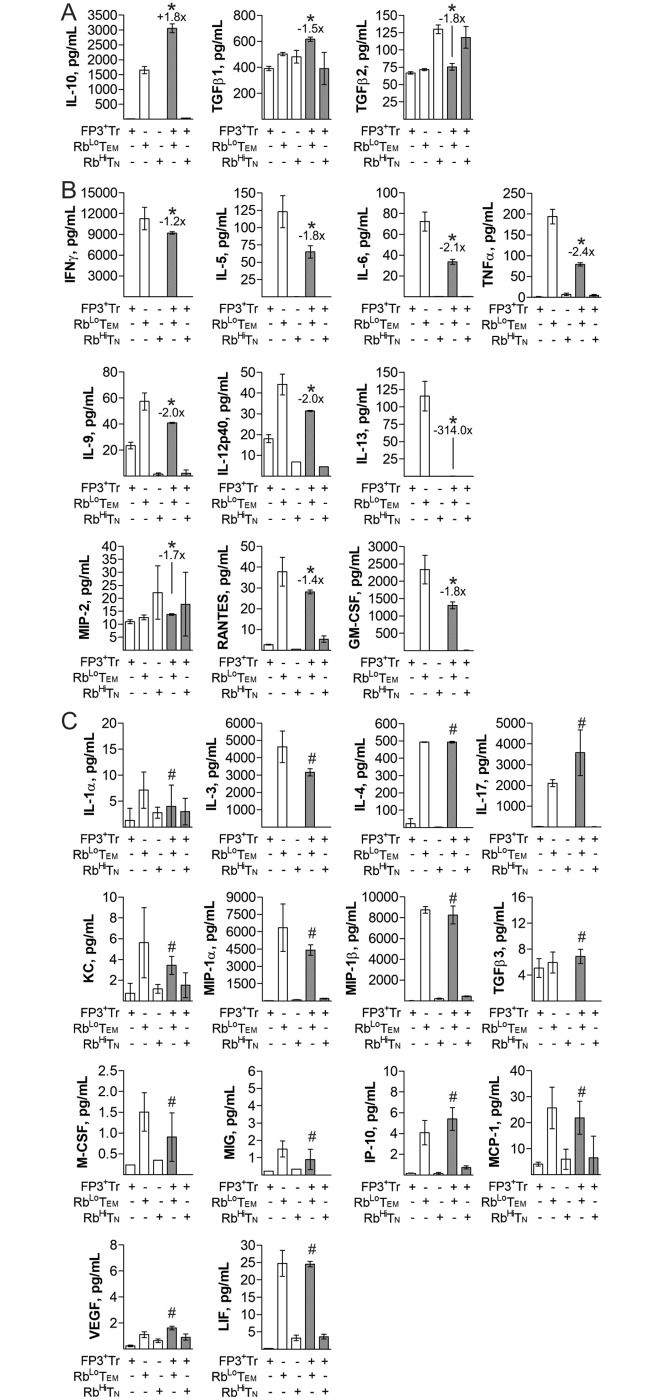
Cytokine synergy of Rb^Lo^Tem and FoxP3^+^ Tregs is limited to IL-10. Rb^Lo^Tem and Rb^Hi^Tn cells were stimulated alone or in co-culture as before for 3 days to determine cytokine and chemokine secretion in relation to the mRNA transcriptomes using multiple Luminex. Statistical comparisons were made based on differences between the sum of the FoxP3^+^ Treg and Rb^Lo^Tem individual responses and the corresponding co-culture. Shown are immune regulation-associated proteins with significant change in co-culture (A), other cytokine and chemokines with statistically significant differences in co-culture (B), and molecules not significantly different in co-culture (C). * = p<0.05; # = p>0.05. P value calculated from Student’s T-Test. Error bars show mean with SEM. n = 3 experiments for all panels.

Although IL-10 is necessary for Rb^Lo^Tem cell-mediated immune suppression [10, 12], we performed multiplex Luminex analysis in order to identify other important cytokines and chemokines. We found that IL-10 was the only cytokine tested that displayed synergistic increases when Rb^Lo^Tem and FoxP3^+^ Tregs were in co-culture (Fig 2A). Two other cytokines closely aligned with immune regulation, TGFβ1 and TGFβ2, were significantly down-regulated in co-culture compared to the combined production of each cell population in isolation (Fig 2A). All other cytokines and chemokines fell into two categories–reduced in co-culture (Fig 2B) or unchanged in co-culture (Fig 2C). Notable molecules with robust decreases included IL-5, IL-6, IL-13, TNFα, and GM-CSF (Fig 2B), while a notable unchanged molecule was IL-4 (Fig 2C).

### IL-10 synergy depends on a soluble mediator

**Fig 3:**
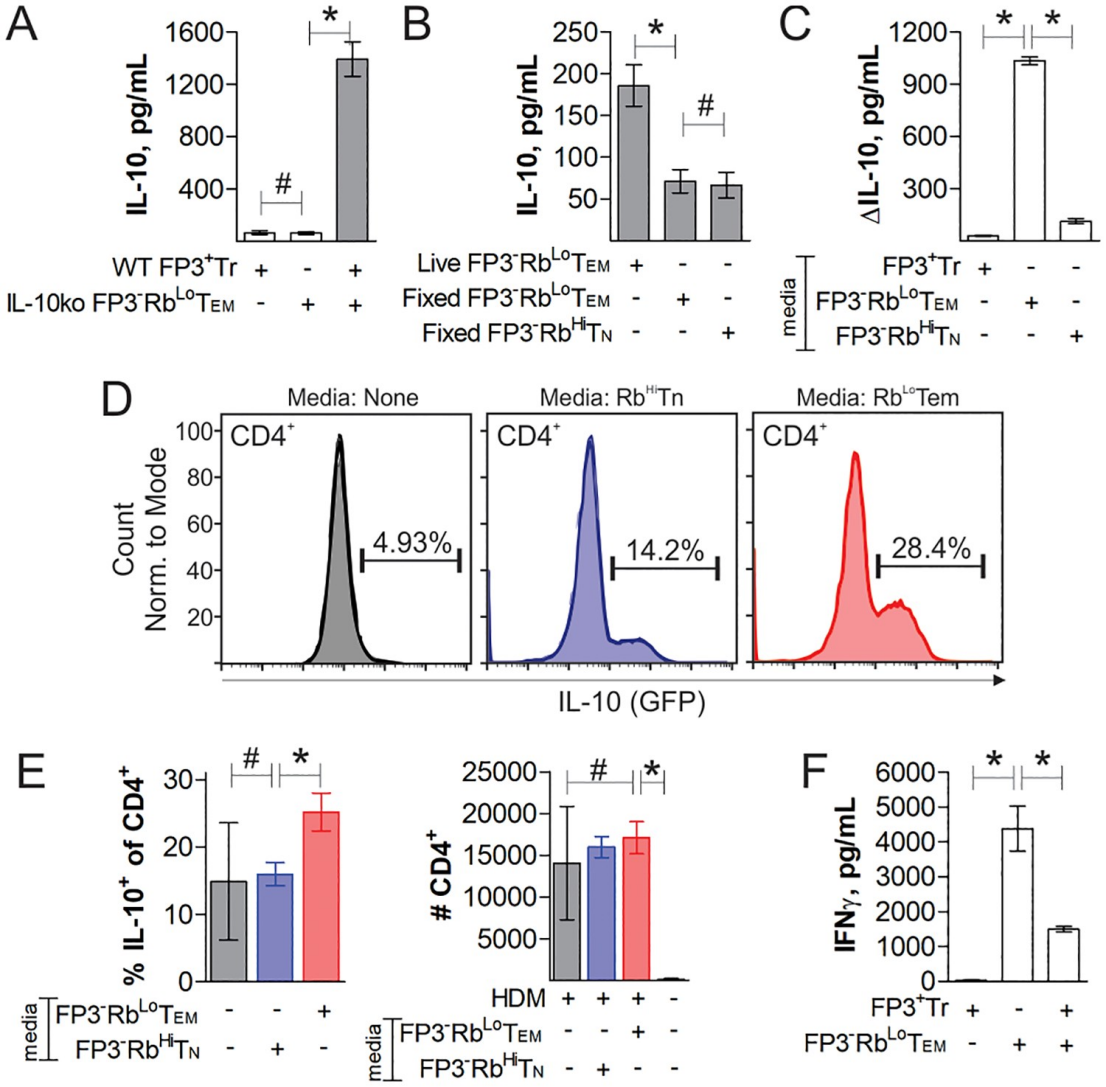
T cell synergy is mediated by a soluble factor. Cells were flow sorted, stimulated with anti-CD3ε antibody for 3 days, and treated as indicated. ELISA measurements of IL-10 concentration in culture supernatant from FoxP3^+^ Tregs co-cultured with IL-10-knockout Rb^Lo^Tem (A), fixed Rb^Lo^Tem or Rb^Hi^Tn cells (B) or the change in IL-10 in the supernatant from cultured FoxP3^+^ Tregs supplemented with conditioned media from Rb^Lo^Tem, Rb^Hi^Tn or FoxP3^+^ Tregs stimulated previously for 3 days as before (C). Conditioned media from activated Rb^Lo^Tem or Rb^Hi^Tn cells were administered to HDM-challenged IL-10-GFP reporter mice intranasally and compared to resting and HDM-challenged mice without conditioned media. Mice were sacrificed and the BALf was analyzed by flow cytometry, showing percent IL-10^+^ among CD4^+^ cells in representative histograms (D) as well as replicates and the total CD4^+^ cell counts (E). ELISA measurements of IFNγ concentration in culture supernatants from FoxP3^+^ Tregs, WT Rb^Lo^Tem, or both cells in co-culture with anti-CD3ε stimulation for 4 days (F). * = p<0.05; # = p>0.05. P value calculated from Student’s T-Test. Error bars show mean with SEM. For A-C, n = 3 to 6 experiments. For E-F, n = 3 experiments.

Our data suggested that Rb^Lo^Tem cells communicate with FoxP3^+^ Tregs to synergistically produce IL-10 (Figs 1 and 2). To confirm that the increase in IL-10 originated from FoxP3^+^ Tregs in these experiments, we cultured Tregs and Rb^Lo^Tem cells alone or together as before, only using Rb^Lo^Tem cells from IL-10-knockout (IL-10ko) mice. ELISA of IL-10 showed that FoxP3^+^ Tregs produce almost no IL-10 when cultured alone, but the inclusion of IL-10ko Rb^Lo^Tem cells dramatically increased IL-10 concentration (Fig 3A). These data show that the Rb^Lo^Tem cells induce FoxP3^+^ Tregs to produce IL-10.

In order to determine the contact dependence of this communication, we measured FoxP3^+^ Treg IL-10 output *in vitro* after co-culturing with either paraformaldehyde-fixed Rb^Lo^Tem or Rb^Hi^Tn cells, or by supplementation of FoxP3^+^ Treg media with conditioned media from stimulated Rb^Lo^Tem or Rb^Hi^Tn cells. We found that fixation of the Rb^Lo^Tem cells eliminated the synergy (Fig 3B), while Rb^Lo^Tem-conditioned media was able to robustly induce IL-10 production in live FoxP3^+^ Tregs (Fig 3C). In addition, neutralizing antibody blockade of the Sema4-Neuropilin cell contact pathway reported to promote Treg activity [22] also did not reduce the synergistic production of IL-10 (S3 Fig). These data suggest that Rb^Lo^Tem cells communicate with Tregs via a soluble mediator(s).

To confirm that conditioned media from Rb^Lo^Tem cells also lead to IL-10 production *in vivo*, we induced pulmonary inflammation in IL-10-GFP reporter mice using lung house dust mite antigen (HDM) challenges and then intranasally administered conditioned media from either Rb^Lo^Tem or Rb^Hi^Tn cells, with untreated HDM mice and resting mice as controls. As seen *in vitro* (Fig 3C), we found that Rb^Lo^Tem but not Rb^Hi^Tn conditioned media significantly increased the percentage of IL-10-producing CD4^+^ T cells in the airway compared to untreated HDM-inflamed mice (Fig 3D and 3E). Although resting mice had too few CD4^+^ T cells for robust analysis of their IL-10 expression pattern, there was no difference in the total number of CD4^+^ T cells in all HDM-challenged mice (Fig 3E). Thus, a soluble mediator(s) produced by Rb^Lo^Tem but not Rb^Hi^Tn cells can induce IL-10 in FoxP3^+^ Tregs *in vitro* and in recipient lung CD4^+^ T cells *in vivo*.

Treg suppressive activity was measured in co-cultures of WT FoxP3^+^ Tregs and WT Rb^Lo^Tem cells for 4 days with anti-CD3ε antibody stimulation. The presence of FoxP3^+^ Tregs significantly reduced the activation state of the Rb^Lo^Tem cells, as measured by IFNγ ELISA, supporting the conclusion that the Tregs are active and suppressive (Fig 3F).

#### The Rb^Lo^Tem transcriptome

**Fig 4:**
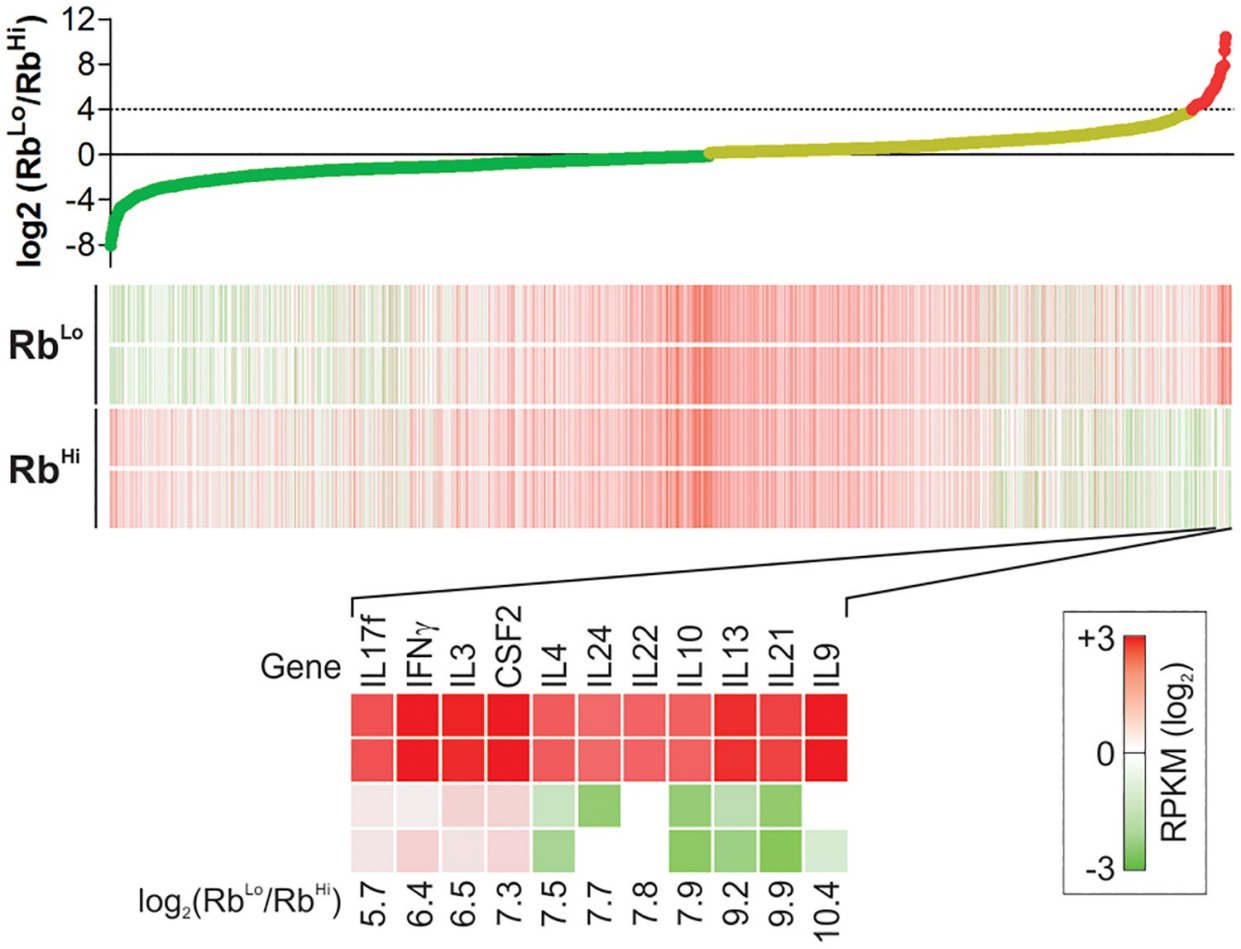
Rb^Lo^Tem and Rb^Hi^Tn transcriptomes reveal potential crosstalk mediators. Rb^Lo^Tem and Rb^Hi^Tn cells were stimulated alone as before for 3 days, and RNA was isolated for deep sequencing and whole transcriptome analysis. Genes were arranged according to the log2 of the Rb^Lo^Tem to Rb^Hi^Tn ratio of expression, using the calculated RPKM values. Candidates were narrowed by excluding all membrane proteins, proteins known to exist only in the nucleus or cytoplasm, and those showing at least 16-fold increase in Rb^Lo^Tem cells compared to Rb^Hi^Tn cells. n = 2 per cell type.

In order to identify the mediator(s), we compared the transcriptomes of anti-CD3ε activated Rb^Lo^Tem and Rb^Hi^Tn cells by mRNA deep sequencing (RNAseq). We arranged the change in copy number by the ratio of Rb^Lo^Tem to Rb^Hi^Tn RPKM values because we predicted that the mediator must be significantly up-regulated in the Rb^Lo^Tem population relative to the Rb^Hi^Tn population. Lead candidates were robustly transcribed (RPKM > 2), at least 16-fold greater in Rb^Lo^Tem over Rb^Hi^Tn cells, and known to be secreted (*i*.*e*., non-membrane proteins; not found in the nucleus or cytoplasm). This analysis revealed a list of highly up-regulated genes which might account for the activity of these cells, including IFNγ, IL-4, IL-22 and others (Fig 4).

#### Neutralization of IL-4 eliminates IL-10 synergy

**Fig 5:**
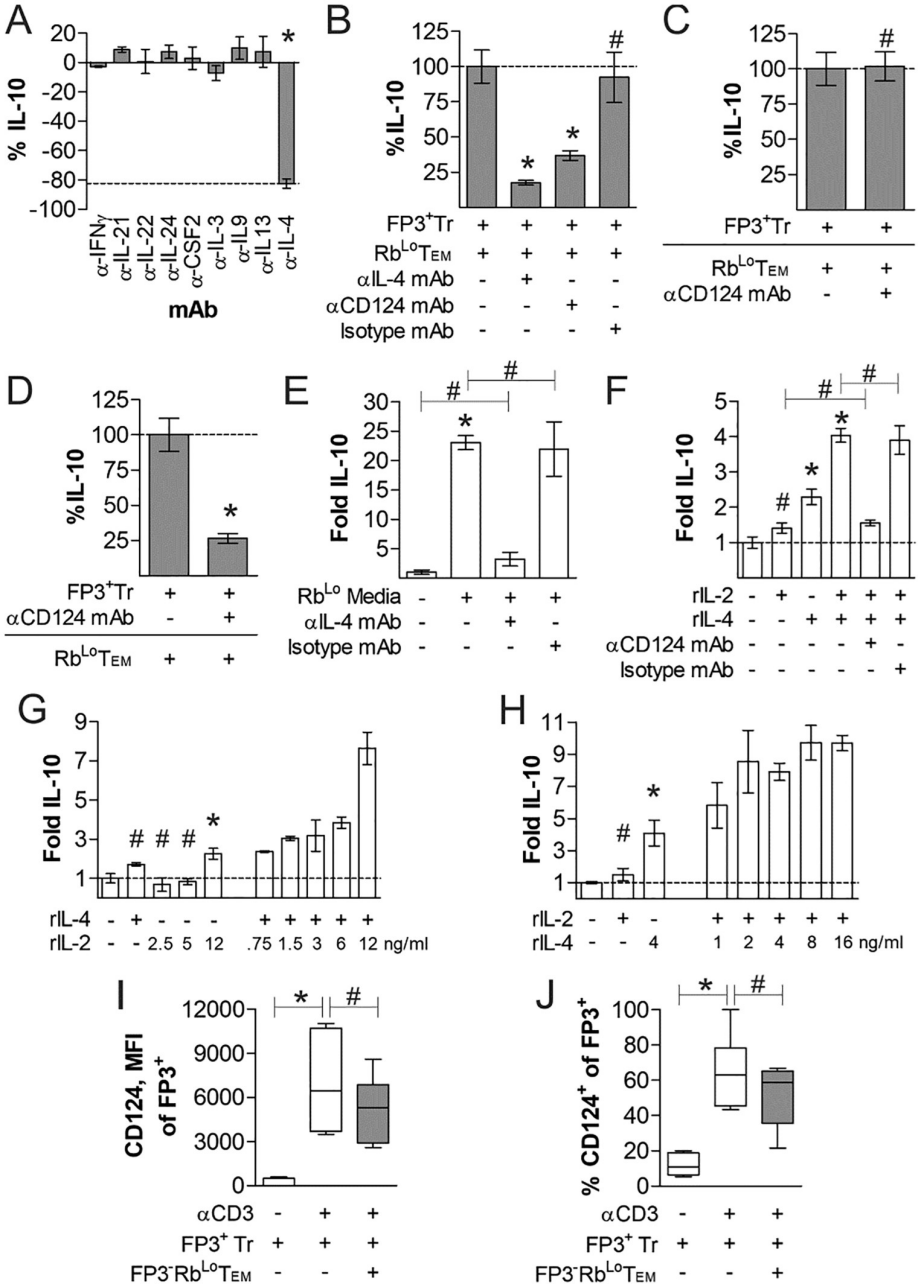
IL-4 and FoxP3^+^ Treg IL-4Rα (CD124) mediates Treg IL-10 production. FoxP3^+^ Tregs and Rb^Lo^Tem were stimulated in co-culture as before for 3 days with the addition of cytokine (A) or receptor (B) neutralizing antibodies to identify the mediator of IL-10 synergy. (C) To determine whether IL-4 was acting on Rb^Lo^Tem cells or FoxP3^+^ Tregs, (C) Rb^Lo^Tem cells were incubated with CD124 neutralizing antibody, washed, then co-cultured with fresh FoxP3^+^ Tregs as before (C); and then compared to pre-incubating FoxP3^+^ Tregs with CD124 antibody followed by co-culture with Rb^Lo^Tem cells (D). FoxP3^+^ Tregs were stimulated as before in monoculture supplemented with either conditioned media from stimulated Rb^Lo^Tem cells (E) or various concentrations, denoted in ng/mL, of recombinant IL-2 or IL-4 (F-H) with and without neutralizing IL-4 or CD124 antibodies (E-H). (I-J) FoxP3^+^ Tregs were cultured with and without αCD3ε stimulation, as well as with and without Rb^Lo^Tem cells. At day 3, cells were harvested and stained for CD124 expression, and analyzed by geometric mean fluorescence intensity (I) and the percent CD124 positive among FoxP3^+^ Tregs (J). * = p<0.05; # = p>0.05. P value calculated from Student’s T-Test, with comparisons to control unless specified. Error bars show mean with SEM. For A-H, n = 3 to 6 experiments. For I-J, n = 6 experiments.

In order to identify the molecules mediating T cell synergy, we performed antibody neutralization experiments during co-culture of FoxP3^+^ Treg and Rb^Lo^Tem cells based on the transcriptomic data (Fig 4). We found that neutralization of IL-4 eliminated the synergistic production of IL-10 (Fig 5A). In repeat experiments comparing neutralization of IL-4 to neutralization of IL-4Rα (CD124), which inhibits both type I and type II IL-4 receptor complexes, we found a corresponding elimination of IL-10 synergy (Fig 5B). To identify which cell the IL-4 was impacting, we neutralized CD124 on only the Rb^Lo^Tem cells by pre-incubation with the antibody, washing away unbounded antibody, then co-culturing the CD124-blocked Rb^Lo^Tem cells with fresh FoxP3^+^ Tregs. We found no difference in IL-10 synergy (Fig 5C). However, the inverse experiment in which the FoxP3^+^ Tregs were pre-blocked with anti-CD124 revealed robust inhibition of synergy (Fig 5D). Moreover, we tested the impact of IL-4 neutralization in FoxP3^+^ Treg mono-cultures using conditioned media from Rb^Lo^Tem cells. Once again, we found that blockade of IL-4 eliminated the IL-10 response (Fig 5E).

In order to determine whether IL-4 was necessary and/or sufficient to induce IL-10 in Tregs, we cultured FoxP3^+^ Tregs with fresh media supplemented with recombinant IL-4 (rIL-4) and IL-2 (rIL-2) together and independently. Although our data already demonstrated that IL-2 was not the primary driver of IL-10 production (Fig 1D & 1F), IL-2 was used because IL-2 concentrations robustly decrease in co-culture (Fig 1D), suggesting that it may play the well-documented pro-survival role for Tregs [23]. We found that rIL-4 induced IL-10 in mono-cultured FoxP3^+^ Tregs, but that the addition of IL-2 greatly enhanced this response despite the failure of IL-2 to robustly induce IL-10 in the absence of IL-4 (Fig 5F). This effect was dependent upon CD124, since neutralizing antibody completely reversed the impact of rIL-4 supplementation (Fig 5F). Cytokine titration experiments with isolated FoxP3^+^ Tregs and varied concentrations of rIL-2 (Fig 5G) and rIL-4 (Fig 5H) further demonstrated the dose-response nature of IL-10 production downstream of exposure to these cytokines. We also found that the expression of CD124 in FoxP3^+^ Tregs was dependent upon prior stimulation with αCD3ε antibody, and this expression was not impacted by the presence of Rb^Lo^Tem cells (Fig 5I and 5J).

### Rb^Lo^Tem cells require IL-4 to protect mice from pulmonary inflammation

**Fig 6:**
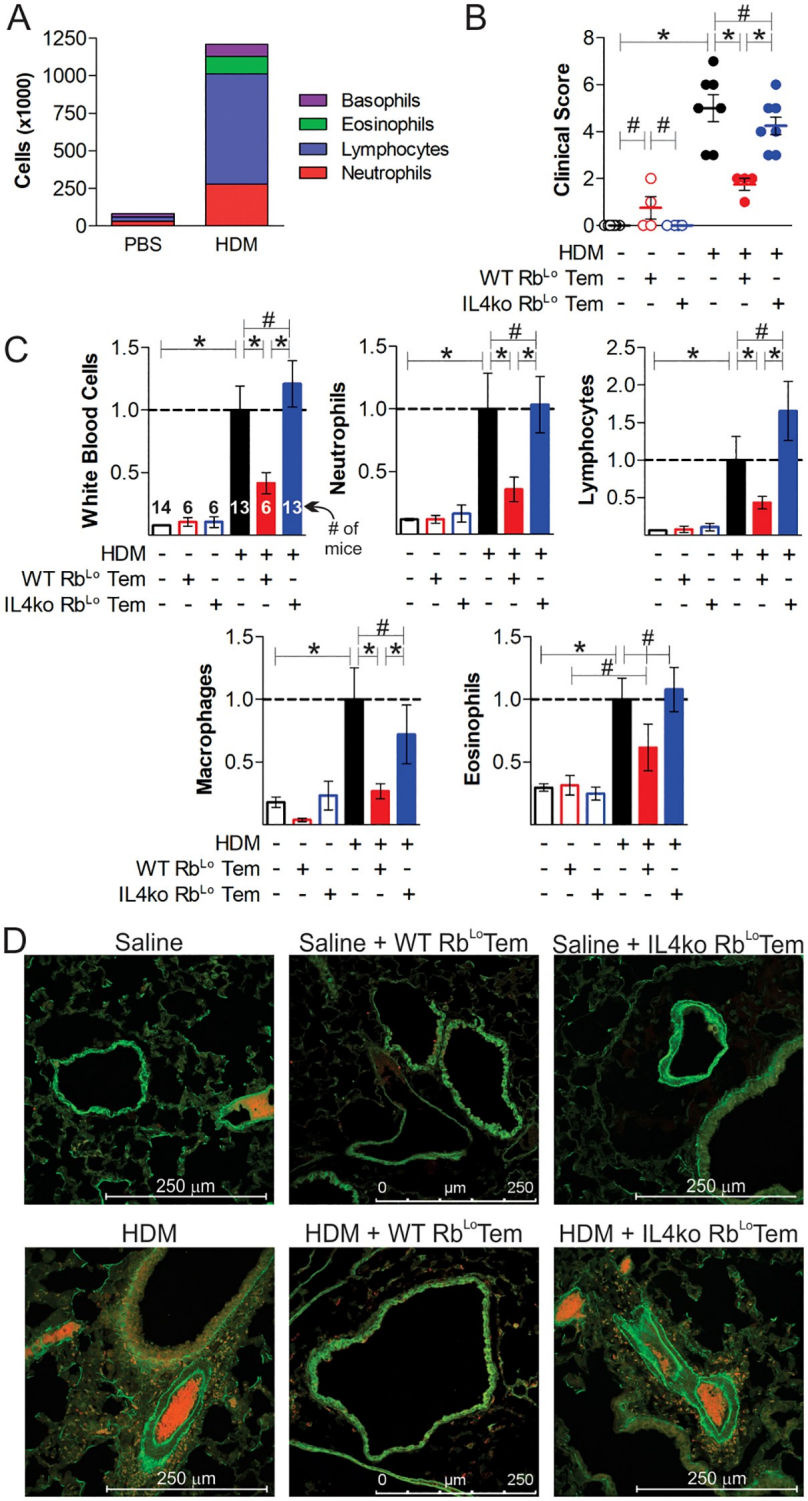
Rb^Lo^Tem cells reverse lung inflammation in an IL-4-dependent fashion. Mice were given 60,000 of anti-CD3ε-stimulated WT or IL-4ko Rb^Lo^Tem cells on day 7 of acute HDM-induced asthma to test their ability to reverse inflammatory pathology. The total number of infiltrating cells (A), the clinical score (see Table 1) (B), normalized cellular differentials from BALf harvests (C), and tissue pathology (D) by immunofluorescence staining for EpCAM (green) and MPO (red) from representative sections of pulmonary tissue are shown. * = p<0.05; # = p>0.05. P value calculated from an unpaired Student’s T-Test. Error bars show mean with SEM. n = 6 to 14 individual mice total per group, across two asthma trials, as indicated on the White Blood Cell counts in panel C.

**Fig 7:**
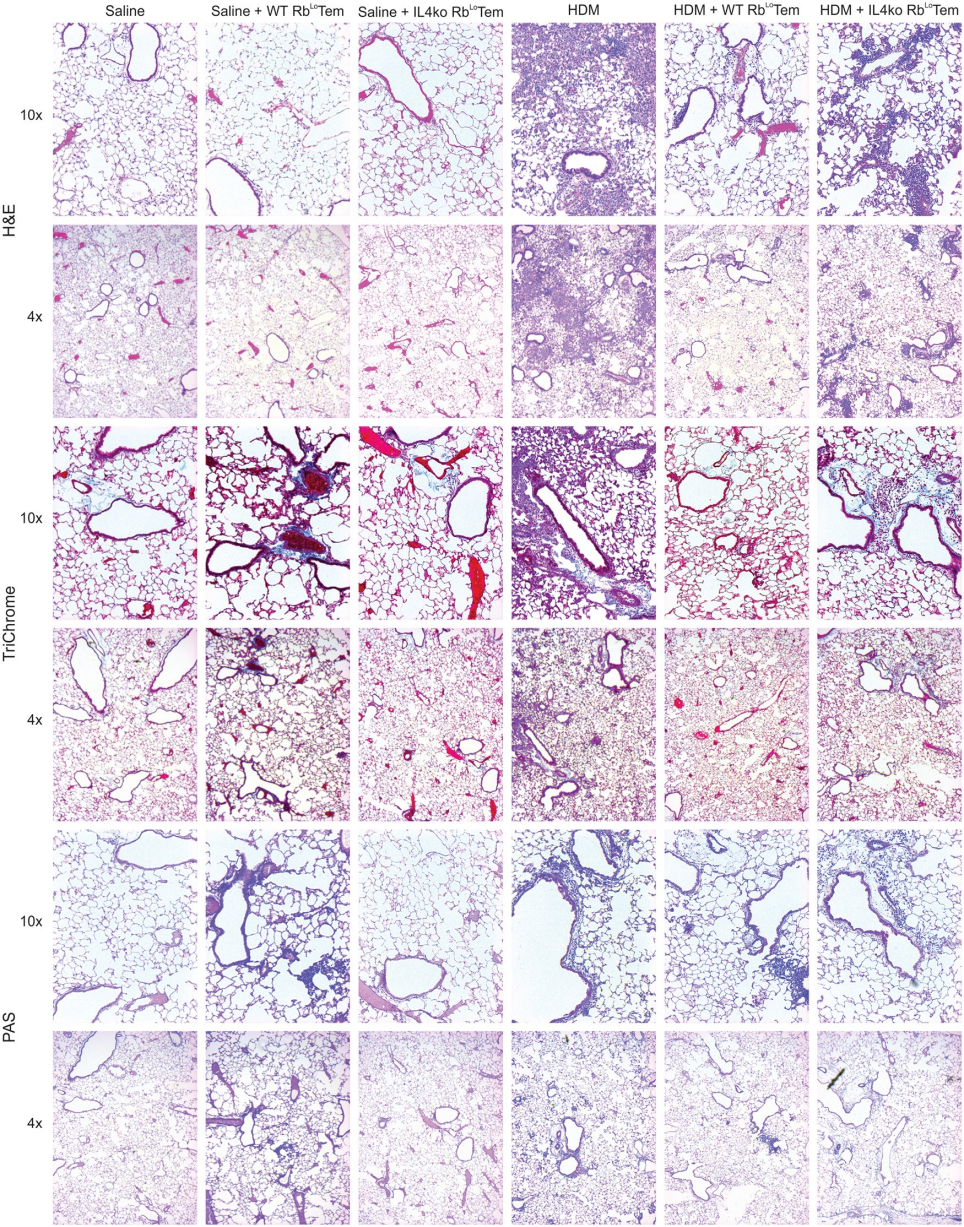
Lung inflammation, remodeling and mucus production is reversed by Rb^Lo^Tem cells in an IL-4-dependent fashion. Representative lung sections from n = 6 mice at 4x and 10x magnification from the mice described in Fig 6 were stained with H&E (top), TriChrome (middle) and PAS (bottom) to assess cellular infiltration, tissue remodeling/collagen deposition and mucus production respectively in HDM-challenged mice receiving 60,000 activated WT or IL-4ko Rb^Lo^Tem cells.

The *in vitro* experiments implicate Rb^Lo^Tem cells as a key population of T cells capable of inducing IL-10 release by FoxP3^+^ Tregs, while transcriptomic and antibody-mediated neutralization experiments implicated a dependence upon IL-4 to promote this response. In order to test the Rb^Lo^Tem protective activity and their dependence on IL-4 *in vivo*, we performed an adoptive transfer experiment into a house dust mite (HDM)-induced acute asthma model. Rb^Lo^Tem cells were isolated as before (S2 Fig) from either WT or IL-4 knockout (IL-4ko) mice, stimulated *in vitro* for 48 hours with anti-CD3ε antibody, then transferred via the tail vein into recipient WT mice on day 7 of HDM challenges. Robust immune suppression, as measured by the total number of airway-infiltrating leukocytes (Fig 6A), overall clinical score (Fig 6B; Table 1), neutrophils, lymphocytes, macrophages and eosinophils (Fig 6C) recovered in bronchoalveolar lavage fluid (BALf), was achieved with a single transfer of 60,000 WT cells. Moreover, tissue pathology measured by confocal microscopy for activated leukocytes (Fig 6D) and hematoxylin and eosin staining (H&E; Fig 7) was returned to baseline in mice receiving WT Rb^Lo^Tem cells, while TriChrome and periodic acid-Schiff (PAS) staining revealed a reversal of collagen deposition (i.e. tissue remodeling) and mucus production, respectively (Fig 7). Importantly, this potent ability to reverse lung inflammation was lost when the Rb^Lo^Tem cells lacked IL-4 (Figs 6 and 7), confirming the central role for IL-4 in Rb^Lo^Tem-mediated immune suppression.

## Discussion

In this study, we discovered a novel pathway of immune suppression mediated by the Rb^Lo^Tem subset of regulatory T cells. We found that FoxP3^-^CD4^+^CD62L^-^CD44^+^CD45Rb^Lo^ effector/memory T cells (Rb^Lo^Tem) coordinate a regulatory circuit in which they communicate with FoxP3^+^ Tregs to synergistically enhance IL-10 production, leading to the suppression of IFNγ and other cytokines *in vitro* and pulmonary inflammation *in vivo*. We also discovered that this pathway is mediated by a combination of IL-2 and IL-4 secreted by Rb^Lo^Tem cells, leading to ligation of IL-4Rα (CD124) on αCD3ε-activated FoxP3^+^ Tregs and subsequent IL-10 production. The discovery of a novel T-effector cell-driven regulatory pathway and its underlying mechanism could have a major impact on how we understand the resolution of inflammation, the maintenance of homeostasis throughout the body, Treg behavior, and possibly how to control a variety of inflammatory diseases.

The interplay between T cell subsets to favor a particular response is not a novel concept. A recent example is the interaction between neuropilin-1 (Nrp1) and semaphorin-4a (Sema4a)[22]. Association of surface Sema4a on Tconv cells with Nrp1 on Tregs increased Treg survival and function *in vitro* and was found to be required for the *in vivo* suppression of inflammatory bowel disease. Here, we found that the direct cell-to-cell contact via the Sema4a-Nrp1 axis does not account for the suppressive influence of Rb^Lo^Tem cells on Tregs, as neutralization of Nrp1 with an antibody had no impact on *in vitro* IL-10 synergy. Instead, our data strongly support a contact-independent mechanism which relies upon soluble mediators, IL-2 and IL-4.

Since the establishment of the T_H_1/T_H_2 paradigm [24–26], it has been widely recognized that T_H_1 responses oppose T_H_2 responses, and vice versa, and that this is driven by key T_H_1 or T_H_2-associated cytokines. IL-4 has long been characterized as a central inducer of T_H_2 cell development [27], a characteristic cytokine made by T_H_2 cells [24–26], and a critical factor leading to the suppression of T_H_1 cell differentiation [27–29]. T_H_2 responses were originally described as including IL-4, IL-5, IL-6, and IL-10 [24–26]. As sophistication in the laboratory setting increased, IL-6 and IL-10 were removed from this general list, but it is pertinent to recall that IL-4 has been well documented to induce IL-10 production in T cells [30]. In fact, IL-4 was linked to anti-inflammatory activity as far back as 1989 [31]. A more recent human trial demonstrated that subcutaneous injection of psoriatic lesions with human IL-4 significantly reduced clinical scores [32], while IL-4 expression from a viral vector was able to prevent chondrocyte death and reduce cartilage erosion in a murine model of collagen-induced arthritis [33]. In EAE, gene delivery of IL-4 into mice selectively recruited FoxP3^+^CD25^+^ Tregs which eliminated the disease pathology [34]. IL-4 has also been implicated in the regulatory mechanisms required for the anti-inflammatory effects of intravenous immunoglobulin [35], and is known to be a robust driver of the wound healing phenotype of many macrophages, which includes IL-10 and TGFβ release [36, 37]. Indeed, IL-4 was previously shown to increase the suppressive capacity of CD25^+^ Tregs *in vitro*, although IL-10 was not assessed at the time [38]. Although it is unclear why IL-4 was not depleted in our experiments (Fig 2) like IL-2 (Fig 1), it may be explained by differences in the relative rates of cytokine release by Rb^Lo^Tem cells and consumption by Tregs, these findings not only show that IL-4 is a highly pleiotropic cytokine with a robust history of immune suppression, but they also strongly support the notion that Rb^Lo^Tem cells could use IL-4 as a key driver of IL-10 and immune suppression under at least some inflammatory conditions.

In terms of asthma, the role of IL-4 is not entirely clear. In early descriptions of asthma one hundred years ago, asthma was divided into “intrinsic” or non-atopic and “extrinsic” atopic disease [39]. However, in the 1980s, the notion that asthma was nearly always atopic came into prominence [40], which lead to the use of asthma as an exemplar T_H_2-mediated atopic disease characterized by T_H_2 cytokines like IL-4, IL-5 and IL-13, eosinophilia and increased IgE production [24–26]. More recent large-cohort studies, such as those conducted by the Severe Asthma Research Program (SARP), have reinvigorated the division of asthma into specific endotypes based on a variety of clinical characteristics. Of these endotypes, it is now widely recognized that atopic asthma is not the only endotype, and that non-atopic asthma accounts for up to 51% of all asthma cases in the US [41].

Consistent with the interpretation that IL-4 is not a central driver of asthma, a number of clinical trials designed to selectively inhibit IL-4 in asthma patients have failed. The trials for a neutralizing anti-IL-4 antibody (Pasolizumab), a soluble IL-4 decoy receptor (Altrakincept), and even a mutant form of IL-4 with a proposed dominant negative impact on IL-4 function (Pitrakinra) all were halted due to a lack of meeting clinical benchmarks of efficacy. In contrast, efforts simultaneously targeting both IL-4 and IL-13 through IL-4Rα blockade have been more successful (Dupliumab), and primarily in eosinophilic asthma [42]. Since IL-13 uses the type II IL-4 receptor, which is comprised of IL-4Rα (CD124) and IL-13Rα (CD213a), the efficacy of Dupliumab supports the notion that IL-13 may be the more important cytokine in terms of asthma pathology. It is also becoming clear that the non-atopic neutrophilic endotypes are more likely driven by a Th17-dominated response, and these patients are much more likely to have greater disease severity and corticosteroid-resistance. Our results using a model of neutrophilic asthma (Fig 6A) fit with the well-known phenomenon that IL-4 opposes Th1 and Th17 responses, and induces IL-10 release in T cells [28, 29].

In summary, our work has revealed a novel T cell-to-T cell regulatory pathway in which IL-2 and IL-4 from Rb^Lo^Tem cells trigger the release of IL-10 in activated FoxP3^+^ Tregs, and that this response can reverse the pathologies associated with HDM-induced neutrophilic lung inflammation in mice. This model fits well into the greater IL-4 literature, implicates a number of additional therapeutic targets within the IL-4Rα pathway which could be explored as clinical therapies, and highlights the myriad of ways in which T cell-mediated responses can be regulated.

## Supporting information

S1 Fig Flow setup for sorting Tregs based on CD25. Experimental design for the gating strategy utilized to capture CD4^+^FoxP3^+^CD25^+^ and CD4^+^FoxP3^+^CD25^-^ cells from magnetically sorted splenocytes from FoxP3-RFP reporter mice.
(TIF)

S2 Fig Flow setup for sorting T cell subpopulations. Experimental design schematic detailing the staining and gating strategy used to isolate CD4^+^FoxP3^+^, CD4^+^FoxP3^-^CD45Rb^Lo^CD44^+^CD62L^-^, and CD4^+^FoxP3^-^CD45Rb^Lo^CD44^+^CD62L^-^ cells from CD4^+^ magnetically sorted splenocytes.
(TIF)

S3 Fig Blocking neuropilin1 does not engage IL-10 production in Tregs. ELISA measurements of IL-10 in mono- and co-culture supernatants after 72h from FoxP3^+^ Tregs or CD4^+^CD45Rb^Lo^ and CD4^+^CD45Rb^Hi^ cells from IL-10n animals. Cells were grown with or without stimulus from plate-bound αCD3ε and supplementation with a blocking αNeuropilin1 antibody. Error bars show mean with SEM.
(TIF)

## Abbreviations

BALf: bronchoalveolar lavage fluid
MHCII: class II major histocompatibility complex
EAE: experimental autoimmune encephalomyelitis
FoxP3^+^: CD4^+^FoxP3^+^ Treg
FoxP3^-^Rb^Hi^: CD4^+^FoxP3^-^CD45Rb^Hi^ T cell
FoxP3^-^Rb^Lo^: CD4^+^FoxP3^-^CD45Rb^Lo^ T cell
GM-CSF: granulocyte/macrophage colony stimulating factor
HDM: house dust mite
IFNγ: interferon gamma
iTregs: inducible Tregs
IL: interleukin
IL-10n: IL-10 null
IL-4ko: IL-4-knockout
IBD: inflammatory bowel disease
MPO: myeloperoxidase
OVA: ovalbumin
PSA: *Bacteroides fragilis* polysaccharide A
RPKM: reads per kilobase of transcript per million mapped reads
Treg: regulatory T cells
Tconv: T conventional (CD25^-^CD4^+^)
Tcm: T central memory (FoxP3^-^CD62L^+^CD44^+^CD4^+^)
Tem: T effector/memory (FoxP3^-^CD62L^-^CD44^+^CD4^+^)
Tn: T naive (FoxP3^-^CD62L^+^CD44^-^CD4^+^)
Rb^Lo^Tem: CD45Rb-low T effector/memory (FoxP3^-^CD62L^-^CD44^+^CD45Rb^Lo^CD4^+^)
T_H_2: type II helper T cell
WT: wild type
