# Supplementary figures and images for: CD45Rb-low effector T cells require IL-4 to induce IL-10 in FoxP3 Tregs and to protect mice from inflammation
Source: PLoS One. 2019 May 23;14(5):e0216893. doi: 10.1371/journal.pone.0216893 (PMC6533033; doi:10.1371/journal.pone.0216893)

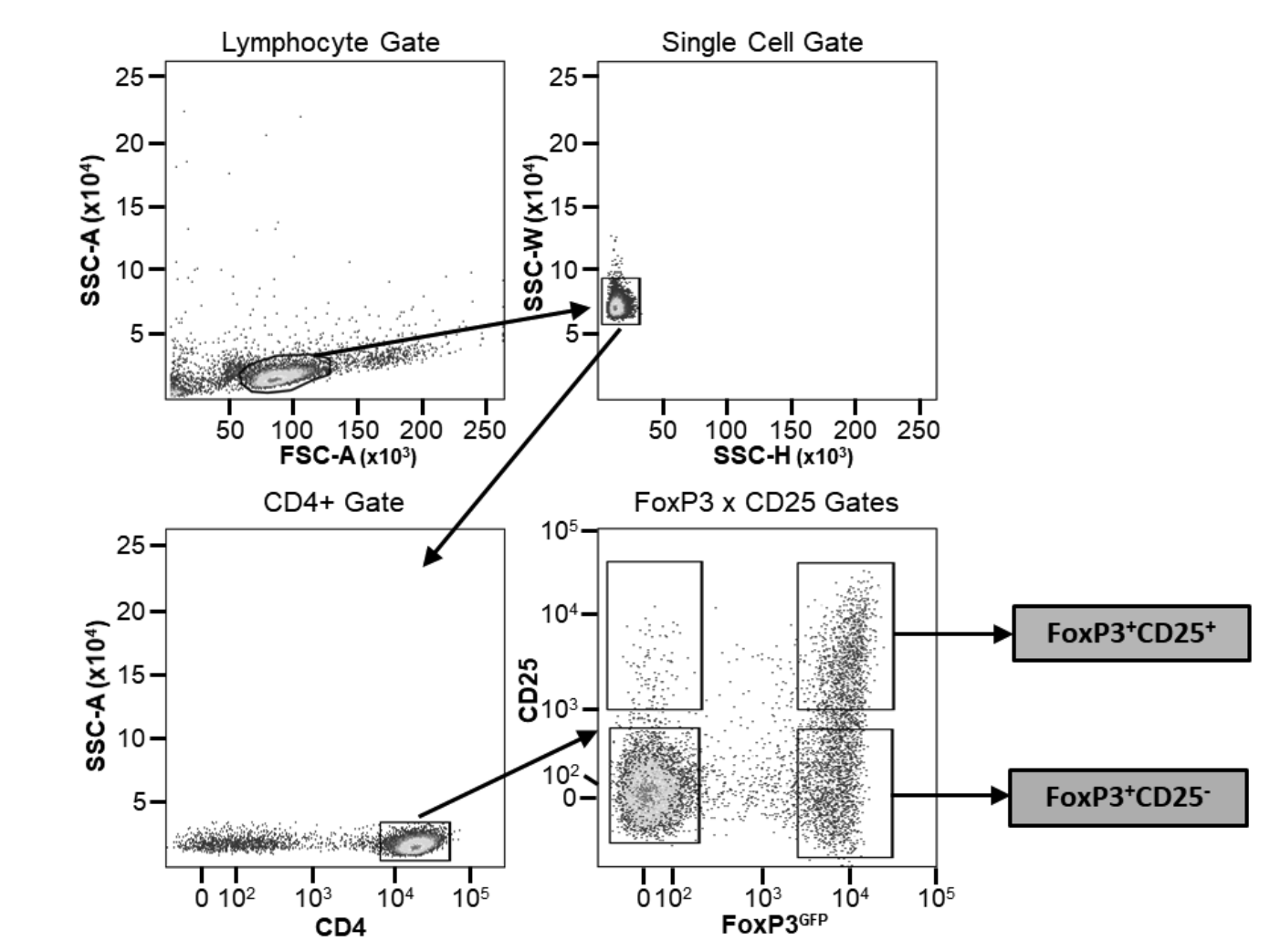

Supplement: S1 Fig — Experimental design for the gating strategy utilized to capture CD4+FoxP3+CD25+ and CD4+FoxP3+CD25- cells from magnetically sorted splenocytes from FoxP3-RFP reporter mice. (TIF) [file pone.0216893.s001.tif]

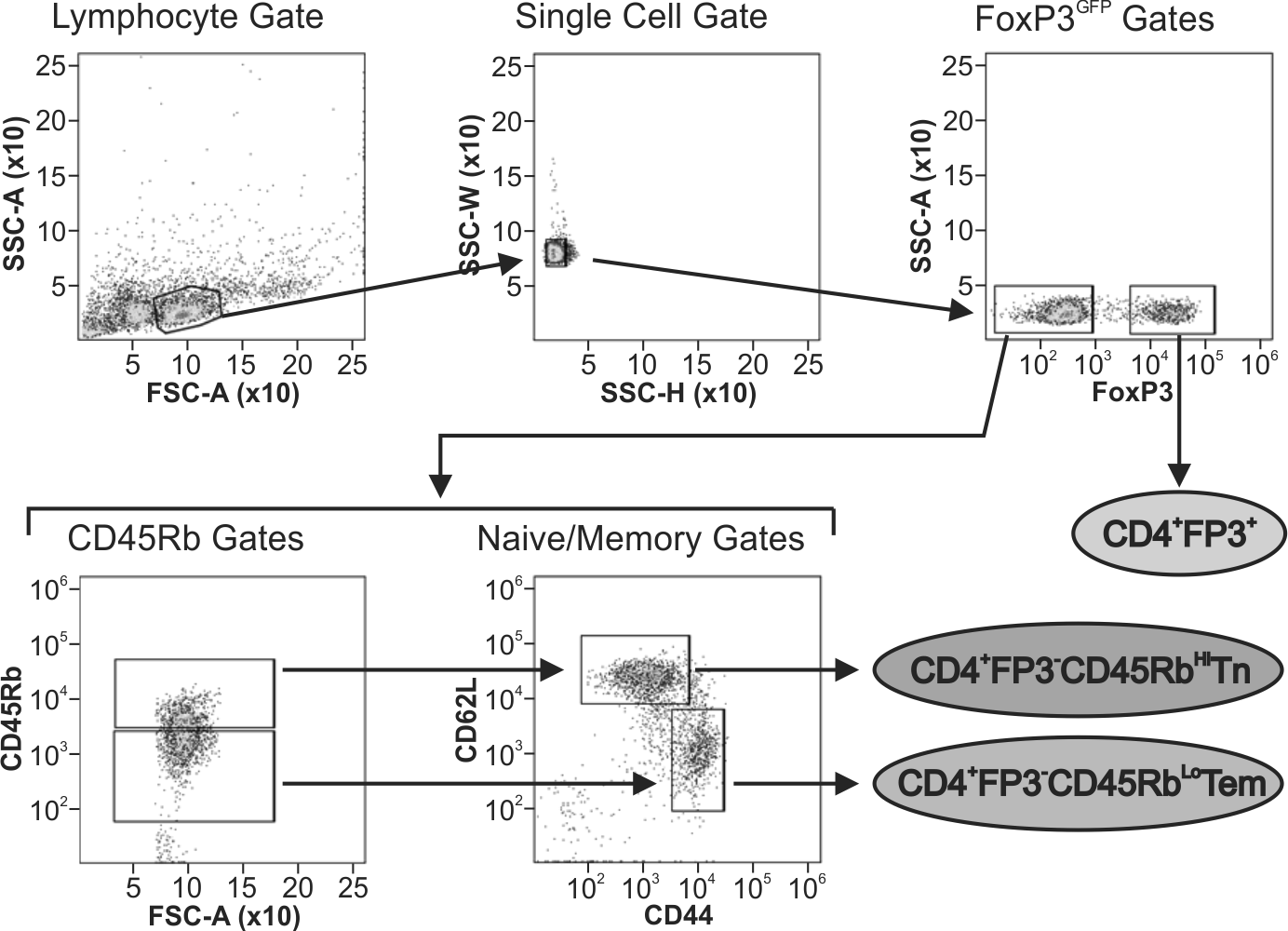

Supplement: S2 Fig — Experimental design schematic detailing the staining and gating strategy used to isolate CD4+FoxP3+, CD4+FoxP3-CD45RbLoCD44+CD62L-, and CD4+FoxP3-CD45RbLoCD44+CD62L- cells from CD4+ magnetically sorted splenocytes. (TIF) [file pone.0216893.s002.tif]

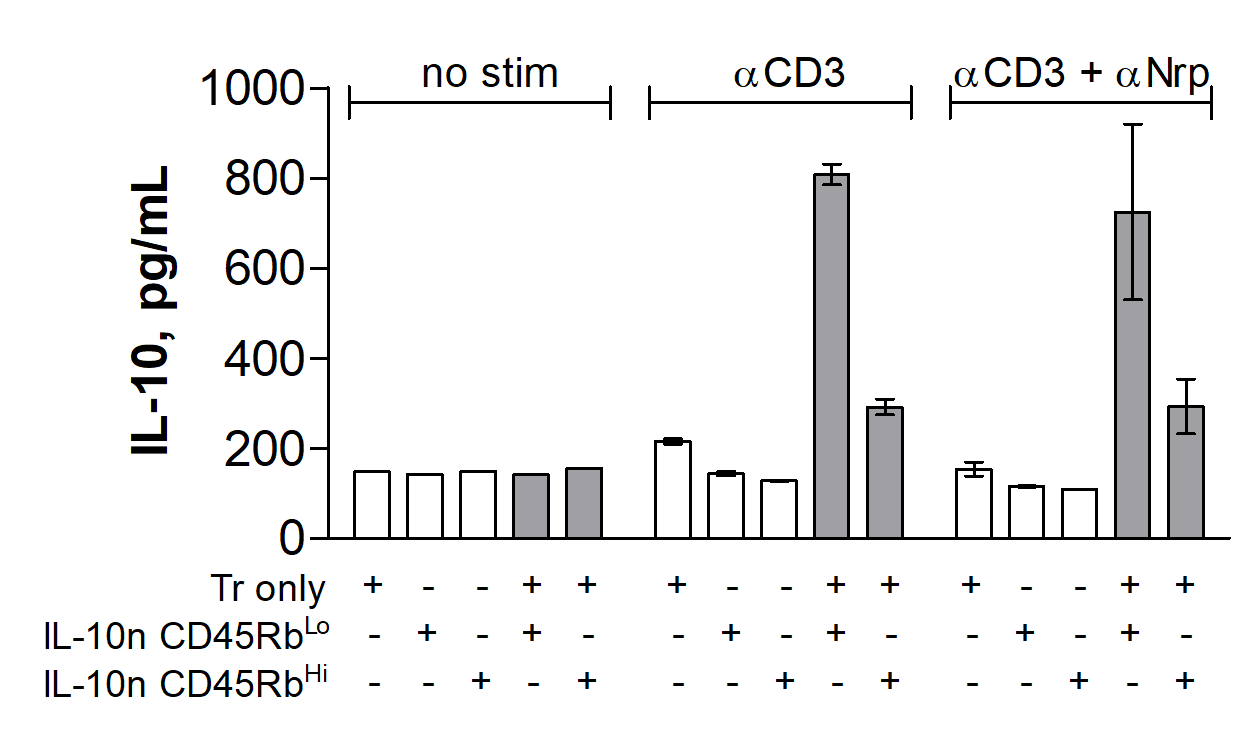

Supplement: S3 Fig — ELISA measurements of IL-10 in mono- and co-culture supernatants after 72h from FoxP3+ Tregs or CD4+CD45RbLo and CD4+CD45RbHi cells from IL-10n animals. Cells were grown with or without stimulus from plate-bound αCD3ε and supplementation with a blocking αNeuropilin1 antibody. Error bars show mean with SEM. (TIF) [file pone.0216893.s003.tif]
